# Challenges and Opportunities Associated With Drug Delivery for the Treatment of Solid Tumors

**DOI:** 10.3389/or.2023.10577

**Published:** 2023-08-30

**Authors:** Teona Paresishvili, Zurab Kakabadze

**Affiliations:** Department of Clinical Anatomy, Tbilisi State Medical University, Tbilisi, Georgia

**Keywords:** extracellular matrix, targeted drug delivery systems, metal nanoparticles, solid tumor, interstitial tumor fluid pressure, tumor vessels

## Abstract

In this review, we discuss the effectiveness of drug delivery system based on metal nanoparticles, and also, describe the problems associated with their delivery to tumor cells. Throughout recent years, more reports have appeared in the literature that demonstrate promising results for the treatment of various types of cancer using metal-based nanoparticles. Due to their unique physical and chemical properties, metal nanoparticles are effectively being used for the delivery of drug to the tumor cells, for cancer diagnosis and treatment. They can also be synthesized allowing the control of size and shape. However, the effectiveness of the metal nanoparticles for cancer treatment largely depends on their stability, biocompatibility, and ability to selectively affect tumor cells after their systemic or local administration. Another major problem associated with metal nanoparticles is their ability to overcome tumor tissue barriers such as atypical blood vessel structure, dense and rigid extracellular matrix, and high pressure of tumor interstitial fluid. The review also describes the design of tumor drug delivery systems that are based on metal nanoparticles. The mechanism of action of metal nanoparticles on cancer cells is also discussed. Considering the therapeutic safety and toxicity of metal nanoparticles, the prospects for their use for future clinical applications are being currently reviewed.

## Introduction

In this review, we draw attention to and discuss the problems associated with the system of targeted drug delivery to the tumor. Despite progress in the development of nanotechnologies, the effectiveness of these systems largely depends on overcoming the barriers created by the tumor microenvironment. It should be emphasized that the effectiveness of targeted drug delivery systems is also related to the structure, size, and shape of nanoparticles (NCs). A web-based search for all types of articles published was initiated using MEDLINE/PubMed (since 1992–2021), with the key words such as “targeted drug delivery systems (TDDS),” “noble metal nanoparticles,” “carriers,” “chemotherapy drugs,” “solid tumor,” “extracellular matrix (ECM),” “interstitial fluid pressure (IFP)” and “tumor vessels.”

The concept of a targeted drug delivery system is somewhat similar to the concept of a “magic bullet” proposed by the German scientist Paul Ehrlich back in 1907 [1]. He suggested that just as a bullet fired from a gun that hits a specific target, a way to target and direct a drug to kill specific disease-causing microbes without harming the body should be found.

Currently, various drug delivery systems are more frequently developed and applied as the new and more promising methods for the treatment of solid tumors. As the authors note, an effective targeted drug delivery systems must fulfil four key requirements: retain, evade, target and release [2, 3].^1^ These requirements can be added the 5R principle used by AstraZeneca: the right target, the right patient, the right tissue, the right safety and the right commercial potential [4].

## Advantages and Disadvantages in Local and Systemic Drug Delivery Systems for Solid Tumor Treatment

Delivery of anticancer drugs to tumor cells is carried out mainly in two ways: by introduction into the systemic circulation or by direct injection into the tumor parenchyma. The administration of anticancer drugs into the systemic circulation is preferable because it is easy to perform and is а better tolerated by patients. Once the anticancer agent is in the blood vessels of the tumor, it penetrates into the interstitium of the tumor through the vascular wall, and spreads by convection or diffusion. The diffusion pathway dominates in tumors of various shapes and sizes [5–7]. However, the method of drug administration through blood vessels is not always effective because the systemic circulation carries anti-cancer drugs throughout the body, which makes it difficult for them to target the tumor, and at the same time, it causes side effects.

It is noted that, that the introduction of antitumor drugs into the systemic circulation leads to the accumulation of low concentrations of drugs at the periphery of the tumor mass near the vasculature without affecting the entire tumor, which can lead to tumor recurrence or metastasis [8–10]. In addition, during transport, the antitumor agents can nonspecifically bind to proteins or other tissue components or be metabolized [11].

Local drug delivery methods are more invasive; however, they are effective in overcoming the potential limitations of systemic transport. The intratumoral or peritumoral injections can increase the retention time of therapeutic drugs in the tumor, induce systemic antitumor responses specific to tumor antigens at the injection site, and thus, can be effective in suppressing tumor recurrence and metastasis potential [12].

## Barriers to the Drug Delivery System to Cancer Cells

### Tumor Blood Vessels

**FIGURE 1:**
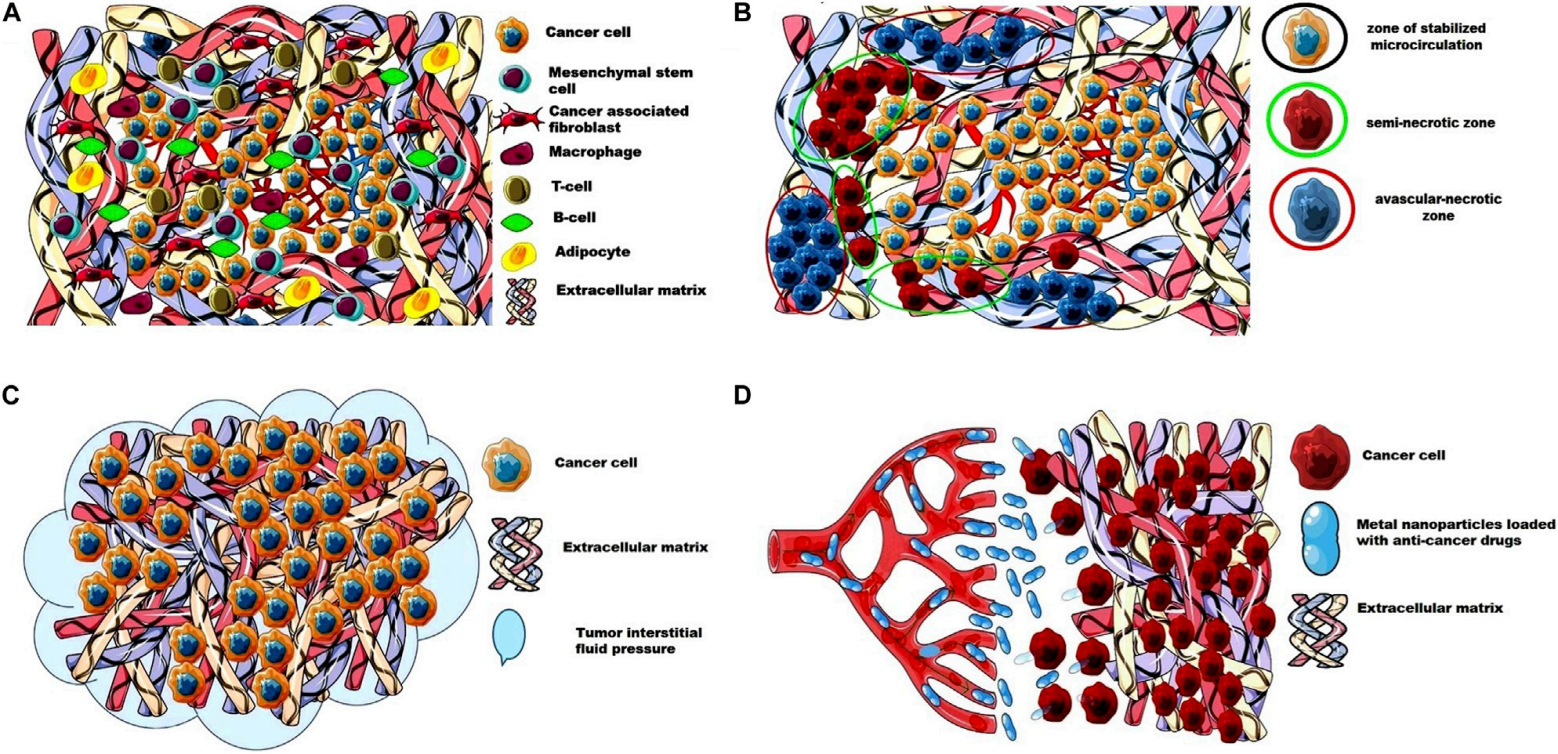
The barriers for the delivery of anticancer drugs to tumor cells. **(A)** Extracellular matrix and cellular composition of a solid tumor; **(B)** Perfusion heterogeneity in solid tumor forms an avascular- necrotic zone, a semi-necrotic zone, and a zone of stabilized microcirculation; **(C)** High tumor interstitial fluid pressure as a barrier for the access of anticancer drugs to tumor cells; **(D)** The dense and stiffer ECM as a barrier for the metal nanoparticles loaded with anti-cancer drugs. This image was produced using images modified from Servier Medical Art^1^, licensed under a Creative Commons Attribution 3.0 Unported License.

In recent years, advances in imaging combined with microscopic techniques have greatly improved our understanding of the structure and angiogenesis of solid tumors. Figure 1 shows the barriers for the delivery of anticancer drugs to tumor cells.

Magnetic resonance imaging (MRI), computed tomography (CT), positron emission tomography (PET), ultrasound and other non-invasive methods allow us to analyze cellular and molecular anomalies in the walls of blood vessels, measure blood flow and vascular permeability, and also identify structural and functional anomalies of the angiogenic blood vessels of the tumor [13]. Unlike the vessels of normal organs and tissues, the vascular system of a solid tumor consists of chaotically arranged tortuous and heterogeneous vessels in their spatial distribution, which have an uneven diameter [14, 15]. Depending on the type of tumor, growth rate, and tumor localization the structure of vasculature can be completely different [16]. In addition, architecture and blood flow can differ significantly between a tumor and its metastases.

The density of blood vessels and the rate of formation of new blood vessels depends on the growth rate and size of the tumor. For example, as the tumor grows the vascular density decreases, resulting in areas of ischemia [17]. There are reports that the tumor blood vessels are more numerous at the tumor-host interface than in the central regions [18]. The blood flow in the tumor is also interesting, which does not follow a constant unidirectional path. As the authors have noted, the tumor vessels are continuously perfused, and within a few minutes, the blood flow can follow different paths or alternate direction through the same vessel [19]. The authors report that perfusion heterogeneity in solid tumors can form an avascular necrotic region, a seminecrotic region, and a stabilized microcirculation region [20]. This perfusion heterogeneity poses problems for the optimal delivery of anticancer agents to all tumor cells. Moreover, large endothelial junctions, an increased number of fenestrations, vesicles, and vesico-vacuolar canals are found in tumor vessels [21–23]. Vascular permeability and hydraulic conductivity of tumors also affect the distribution of anticancer drugs in the tumor parenchyma. It should also be mentioned that vascular permeability can be different within the same tumor and it depends on the rate of tumor growth, regression or recurrence [24, 25], as well as on the host production of cytokines such as vascular permeability factor (VPF)/VEGF and its inhibitors [26, 27].

Various strategies have been proposed to overcome the barriers that have arisen in the way of drug delivery to cancer cells. Some strategies are based on the use of anti-angiogenic agents (AIs), which prevent the formation of new blood vessels, vascular disrupting agents (VDA) and agents that restore the altered vessels of the tumor [28–31]. Other strategies include physical (radiation, heat) and chemical (vasoactive drugs) methods or usage of pH-Sensitive biomaterials, which can lead to increased blood flow in the tumor or can increase their permeability [32–35].

However, most of these strategies have certain disadvantages. The use of angiogenesis inhibitors, especially in combination with chemotherapy, has been reported to be associated with toxicity due to systemic disruption of growth factor signaling pathways that mediate their anti-angiogenic activity [36, 37], with severe or fatal bleeding [38] and with arterial hypertension [39]. There are also reports that VEGF-targeted therapy induces a period of stable disease followed by VEGF-independent vascular growth promoting cell invasion and metastasis [40, 41].

The advent of VDA has greatly improved the treatment of solid tumors by causing a rapid and selective cessation of blood flow in the tumor. However, when using VDA, residual cells may remain viable at the edge of the tumor, which is the main reason for recurrence [42]. In addition, most vascular disruption agents have large volumes of distribution, shorter half-life, and cardiotoxicity after systemic administration [43].

### Tumor Extracellular Matrix

The extracellular matrix (ECM) in mature normal tissues is a structurally stable composite consisting of porous three-dimensional structures of collagen, proteoglycan, elastin macromolecules and cell-binding glycoprotein. Each of these components has different physical and biochemical properties. Collagen provides the structural and mechanical integrity of the ECM, while proteoglycans regulate the movement of fluid and solutes [44]. The ECM is also a rich source of growth factors and bioactive molecules and is actively involved in cell proliferation, adhesion, migration, polarity, differentiation, and apoptosis [45, 46]. It should be noted that the ECM is closely associated with the basement membrane, which may represent a certain form of the ECM itself [47]. The basement membrane, composed of collagen IV and laminins, is a dense structure that divides tissues into well-organized compartments [48]. Binding of cells to the basement membrane is necessary to establish the polarity of epithelial cells [49]. However, basement membrane ECM of the tumor vasculature is more porous and leaky and promotes tumor cell metastasis [50].

In recent years, the ECM of solid tumors has been of particular interest to researchers as one of the barriers that prevent the transport and delivery of anticancer drugs to cancer cells. As the authors note, 60% of the mass of a solid tumor is the ECM, which is a dense and stiffer structure [51]. In this article, we will not discuss the mechanisms of tumorigenic remodeling of the ECM. They are described in detail by many authors [52–55]. We only note that, excessive accumulation of dense and stiffer ECM encapsulating tumor cells can impair the diffusion of oxygen, nutrients and metabolites, leading to hypoxia and metabolic stress. Moreover, increased hypoxia and metabolic stress lead to the activation of anti-apoptotic pathways and drug resistance of the tumor [56]. Our attention focused on the problems associated with the interstitial transport of anticancer drugs in the tumor parenchyma. The study of the authors concerning evaluation of interstitial transport of IgG and BSA proteins in four different tumor lines (human colon adenocarcinoma, LS174T; human glioblastoma, U87; human soft tissue sarcoma, HSTS 26T) is of particular interest; all of them were xenotransplanted in Nude mice NCr/Sed-nu/nu and murine mammary carcinoma (MCaIV) reared in C3H mice [57]. The authors conclude that solid tumor ECM can be thought of as a dispersion filter that controls the composition of the extracellular fluid and the rate of molecular transport. Collagen and proteoglycans play one of the main roles in this process. It was also noted that the delivery of macromolecular agents is facilitated in tumors with poorly organized and weakly interconnected collagen networks.

We agree that the ECM of solid tumors represents a barrier to drug migration and is the cause of failure of many cancer treatments. To solve this problem, various ways are proposed for optimal delivery of anticancer drugs to tumor cells by destroying the ECM of the tumor. Enzymes such as collagenase [58] or pegylated hyaluronidase [59, 60] are used as agents for the destruction of the extracellular matrix of the tumor. The authors note that hyaluronidase digests the ECM, providing easy diffusion of drug molecules to the target. It is also suggested that they act on cancer-associated fibroblasts, which significantly reduce the deposits of ECM [61]. The structure of collagen in the extracellular matrix of solid tumors can be destroyed by high-intensity pulsed focused ultrasound (HIFU). As the authors note, HIFU can not only destroy the collagen structure, but can also be used to induce local hyperthermia [62, 63].

An interesting strategy is to modulate the ECM of solid tumors using small molecules. It is reported that therapeutic targets of the ECM can be thrombospondins, osteopontins, periostins, tenascins, matrix metalloproteinases, and cathepsins [64–66], as well as matrix metalloproteinases (MMPs), a zinc-dependent family of proteinases which is most implicated in matrix degradation [67]. Proteins, glycoproteins and proteoglycans can also be therapeutic targets.

In summary, а better understanding of the biology of the ECM of solid tumors, and the development of effective methods for its destruction will provide an opportunity to significantly improve the results of cancer treatment.

### High Tumor Interstitial Fluid Pressure (TIFP)

High tumor interstitial fluid pressure (TIFP) can be considered one of the hallmarks of almost all solid tumors. TIFP is the result of abnormal cancer cell proliferation, dense and stiffer ECM, leaky and immature blood vessels, and the absence of normally functioning lymphatic vessels. In addition, TIFPs prevent the transport of anticancer agents to all tumor cells, causing their heterogeneous distribution in the tumor mass [68]. As the authors have noted, if the interstitial fluid pressure (IFP) in the normal tissues ranges from −3 to 3 mmHg, then, the TIFP in various tumors ranges from 5 to 40 mmHg and above, reaching 100 mmHg [69]. It has also been reported, that TIFP increases with tumor volume [70, 71]. For example, a comparative assessment of the IFP of a ≥3 cm breast tumor and the IFP of normal tissue of the same breast showed a significant difference. According to the authors, the mean and median values between the initial level of IFP in normal tissue and tumor IFP were 1.05 and 6.5 mmHg, respectively [72]. In patients with cervical cancer, the mean IFP was 19 mmHg, and in patients with intracranial tumors, the mean IFP was 2.0 ± 2.5 mmHg [73, 74]. High TIFP induces fluid flow from the center of high pressure to the periphery of the tumor and thus, prevents effective delivery of anticancer drugs to tumor cells [75]. The mechanisms of accumulation of fluid in the interstitium of the tumor are described in detail by many authors [76]. To reduce TIFP in solid tumors, various strategies are being developed including: normalization of the integrity of tumor vessels using antibodies against VEGF in combination with cytotoxic therapy [22, 77]. However, strategies aimed at reducing TIFP in tumors, require more detailed studies to improve their effectiveness [78–82]. Our attention was drawn to a method of delivering drugs to head tumors using the Ommaya reservoir [83]. Using the principle of the Ommai reservoir, we have developed a mini-catheter to reduce pressure in the tumor and deliver anticancer drugs to solid tumor cells [84]. The conducted experimental studies have shown the promise of this method for the treatment of solid tumors.

## Careers for Drug Delivery Based on Metal Nanoparticles

Another problem associated with the targeted drug delivery of the anticancer drugs to the tumor cells is the search for an ideal carriers that can penetrate the solid tumor parenchyma and deliver these drugs to all regions of the tumor. However, the choice of such a drug carrier is still a matter of debate. Some authors report that an ideal carrier for targeted drug delivery systems should have targeted effects, have a sufficiently strong adsorption effect for antitumor drugs, and release drugs from them at sites that are relevant in effect [85, 86].

Most drug carriers are NPs derived from inorganic and organic materials, and from synthetic polymers as well. In recent years, submicron particles for drug transport have been developed, including мicelles, сubosomes and hexasomes, liposomes, lipid NPs, nanoemulsions, polymer-based self-assemblies, etc. [87–93]. The authors report that in passive targeting, because of the enhanced permeability and retention (EPR) effect, the macromolecules including NPs accumulate preferentially in the neoplastic tissues [94, 95]. Other authors have reported that the effect of EPR provided a rather modest tumor specificity with a 20%–30% increase in delivery compared to normal organs [96]. It is noticed that the effect of EPR strongly depends on the degree of angiogenesis and lymphangiogenesis, the degree of perivascular tumor growth and the density of the stromal response, and intratumoral pressure [97]. Two strategies are used to improve the accumulation of NPs in a tumor with active targeting: the strategy of target molecules that can endow nanosystems with purposefulness and, the strategy of modulating the protein crown of nanocarriers to provide “natural targeting” to the tumor microenvironment (TME) [98–100].

It is known that metal NPs have unique physiochemical properties which allows them to be used for drug delivery, diagnosis and treatment of cancer. They can be synthesized by changing size and shape. According to the authors, the control in particle size in conjugation with surface coating with stealth ligand allows them to veil against body’s immune system and circulate in the blood for longer period of time [101]. The effectiveness of their use depends on the stability of metal NPs, their biocompatibility and the ability to selectively target the tumor tissue after their systemic or local administration. It is reported that one can obtain more specific targeting systems for recognizing cancer cells by conjugating a metal NP with an appropriate ligand. Also, the authors report that the surface of the NPs is able to attach multiple copies of the chemotherapeutic drug and thus, increase the concentration of both therapeutic and diagnostic substances in the pathological site [102].

### Design of Drug Delivery Systems Based on Metal NPs

The design of the drug delivery systems on the basis of metal NPs is actively discussed in the literature. Some authors report that the most optimal size of NPs capable of collecting inside the tumor tissues is approximately 100 nm [103]. Other authors think that the smallest NPs of <20 nm can penetrate deep into the tumor tissue, and NPs of >100 nm in size are positioned within the blood vessel since they remain locked in the extracellular matrix between the cells [2, 104]. As for the shape of NPs, the authors report that spherical or cubic NPs have the highest rate of intracellular internalization, while disk-shaped or rod-shaped NPs show the lowest rate [105].

The circulation time of NPs, their penetration speed and intracellular internalization can also depend on the surface charge of NPs. The studies conducted by the authors have shown that all cancer cells in any organs have a negative charge [106]. They have reported that the cancer cells bind strongly to the positively charged nanoprobes while normal cells, regardless of the probe’s positive or negative charge, bind insignificantly. Thus, it can be presumed that positively charged NPs can achieve higher cellular interaction and absorption by the cancer cells that possess a negative surface charge.

The process of converting a neutral/negative charge into positive charge depend on the change of chemical structure of nanocarriers such as protonation/deprotonation, bond breakage, and change of molecular structure [107]. These processes can be triggered by the internal or external specific stimuli such as pH, redox potential, enzymes, light or temperature.

In this regard, the works related to the use of an intelligent dual pH-responsive self-aggregating nano gold system (Au@PAH-Pt/DMMA) for the combined chemo-radiotherapy seems to be interesting, in which a “charge-reversal like” strategy is utilized to realize irreversible stable aggregation and pH-specific release of cisplatin prodrug in TME [108]. The authors report that the dual pH-responsive NPs Au@PAH-Pt/DMMA could effectively enhance anti-tumor therapeutic efficiency by combined chemo-radiotherapy, which provides a potential method for clinical transformation of cancer treatment.

### Gold Nanoparticles (AuNPs)

The gold NPs are recognized as the attractive candidates for drug delivery to tumor cells and are being investigated as photothermal agents, contrast agents and radiosensitisers [109].

Interest was instigated by the publication of the authors related to the use of the delivery system containing gold NP as a delivery vehicle, cetuximab as a targeting agent, and gemcitabine as an anticancer drug [110]. As the authors report *in vitro* targeting efficacy tested against three pancreatic cancer cell lines (PANC-1, AsPC-1, and MIA Paca2) with variable epidermal growth factor receptor (EGFR) expression, and showed that gold uptake correlated with EGFR expression. The *in vivo* study further confirmed that the inhibition of tumor growth was due to targeted delivery.

Other authors have used AuMSS nanorods that were dual-functionalized with Polyethylene glycol methyl ether (PEG-CH3) and Gelatin (GEL) to enhance both the colloidal stability and uptake by HeLa cancer cells. Additionally, the AuMSS nanorods were combined with IR780 (a heptamethine cyanine molecule) [111]. The results have shown that the combination of photodynamic and photothermal therapy mediated by IR780-loaded AuMSS/T-PEG-CH3/T-GEL nanorods effectively promote the ablation of HeLa cancer cells.

According to the authors, “Anti-HER2-functionalized gold nanoshells on silica” have been shown to target HER2-positive breast cancer cells [112].

The authors reported on the use of orally absorbable gold nanoparticles (AuNP) to treat glioblastoma multiforme (GBM) for patients with its highest incidence rate [113]. They used a milk protein lactoferrin-conjugated AuNP for its oral absorption and targeting to the GBM through the interaction between lactoferrin (Lf) and lactoferrin receptor (LfR) that is highly expressed in the intestine, blood-brain barrier and GBM. Glutathione and polyethylene glycol (PEG) was injected for the stability and long circulation of AuNP. The authors note that orally administered Lf-PEG-AuNP exhibit an outstanding temperature rise in GBM by irradiating laser and significantly reduce tumor volume. They also assume that the Lf-PEG-AuNP can fundamentally target GBM in the brain through oral absorption, and that its efficient photothermal therapy is possible. For combined chemo-photothermal therapy of colorectal cancer, 7-ethyl-10-hydroxycamptothecin (SN-38) loaded with gold nanoparticles (HSP@Au NPs) were used [114]. As the authors report, the HSP@Au NP-mediated chemo-photothermal therapy displayed significant tumor growth suppression and disappearance (25% of tumor clearance rate) without adverse side effects *in vivo*.

### Platinum Nanoparticles (PtNPs)

For many decades, the platinum-based anticancer drugs have been widely used as first-line drugs in cancer chemotherapy for various solid tumors. For example, cisplatin is still used in standard chemotherapy regimens. However, its use is often associated with severe systemic toxicity, especially after long-term treatment. Platinum-based anticancer drugs such as carboplatin, oxaliplatin, nedaplatin, and others can also cause side effects [115].

The interest of researchers in platinum NPs is due to the fact that due to passive targeting, the NPs preferentially accumulate at the tumor site, and the addition of tumor-targeting fragments further enhances their tumor-specific localization, as well as absorption by tumor cells [116].

As reported by the authors, the content of platinum in DNA cells of human colon carcinoma (HT29) increases depending on time and concentration with a maximum effect at 1,000 ng/cm^2^. They suggested that DNA strand breaks mediated by metal Pt-NPs are caused by Pt ions formed during cell incubation with these NPs [117].

In recent years, the synthesis of new prodrugs based on Pt(IV) can minimize off-target interactions and side effects on healthy cells. Pt (IV) complexes act as prodrugs that are activated inside cancer cells releasing cytotoxic Pt (II) drugs such as cisplatin [118].

For example, a prodrug representing the synthesis of (OC-6-44) acetatodiamminedichlorido [2- (2-propynyl) octanoato] platinum (IV) which they called Pt(IV) Ac-POA was developed for the treatment of glioblastoma [119]. As reported by the authors, Pt (IV)Ac-POA was able to induce tumor cell death at low concentrations, demonstrating a persistent antitumor effect that persists with long-term treatment. Other authors developed a novel carrier, micelle-type bioconjugated PLGA-4-arm-PEG branched polymeric NPs, for the detection and treatment of pancreatic cancer [120]. The authors note that the prepared polymeric NPs may serve as a promising platform for the detection and targeted drug delivery for pancreatic cancer. Our special interest is drawn towards the hydrogels containing platinum NPs and the possibility of their use for the treatment of tumors.

An injectable and degradable photothermal hydrogel encapsulated in a platinum NP dendrimer (DEPts) cross-linked with aldehyde-modified dextran via imine bond formation has been reported [121]. The results of the study showed that after the treatment of the tumor, the hydrogel gradually resorbed due to the destruction of imine bonds, which led to complete regression of the tumor. The development of a biodegradable thermosensitive copolymer hydrogel for co-delivery of the antitumor agent gemcitabine and cisplatin has also been reported [122]. As the authors noted, compared to an intravenously administered free combination of gemcitabine and cisplatin, a single intratumoral injection of the two-component hydrogel formulation demonstrated superior antitumor efficacy and minimized systemic side effects in a mouse colonic pancreas xenograft model.

In 2020, we presented a two-layer fibrin-based multicomponent gel (MCPFTG) for the prevention of recurrence and metastases after tumor resection [123]. Our studies have shown that the MCPFTG-based local drug delivery system effectively suppresses residual tumor cells and prevents recurrence.

## Discussion

With the rapid development of nanotechnologies, the prospect of using metal nanoparticles for an early diagnosis of the localization of tumor lesions and cancer treatment has appeared. Metal nanoparticles can be loaded with various antitumor drugs to create targeted drug delivery systems. However, the use of metal nanoparticles in clinical practice is accompanied by many problems, the main of which is their toxicity, which can be different depending on the specific nanoproperties of the metal. Another problem is delivery of anticancer drugs to tumor cells. As you know, the delivery of anticancer drugs is carried out mainly in two ways: by introduction into the systemic circulation or by direct injection into the tumor parenchyma. The administration of anticancer drugs into the systemic circulation is preferable because it is easy to perform and is а better tolerated by patients. However, this method is not always effective because the systemic circulation carries anti-cancer drugs throughout the body, which makes it difficult for them to target the tumor, and at the same time, it causes side effects. The intratumoral or peritumoral injections can increase the retention time of therapeutic drugs in the tumor, induce systemic antitumor responses specific to tumor antigens at the injection site, and thus, can be effective in suppressing tumor recurrence and metastasis potential. However, when administered intratumorally, the effectiveness of nanoparticles will be related to their ability to overcome tumor tissue barriers such as the atypical structure of blood vessels, dense and rigid extracellular matrix, and high pressure of the tumor interstitial fluid. Various strategies have been proposed to overcome these barriers, including the use of anti-angiogenic agents, which prevent the formation of new blood vessels, various enzymes for destroying the extracellular matrix of the tumor and others. However, most of these strategies have certain disadvantages and require further detailed studies.

Currently, there are over 50 drugs based on NPs that have been approved for clinical use by both the Food and Drug Administration (FDA), United States, and the European Medicines Agency (EMA). Additionally, more than 30 drugs that are based on NPs are undergoing various phases of clinical trials [124, 125]. Clinical studies have shown the promise of using NPs that are based on liposomes for the drug delivery to the tumor [126, 127]. The clinical studies of anticancer drugs that are based on gold and platinum NPs are also being conducted [128, 129].

Based on this review, it was concluded that some metal nanoparticles such as platinum, due to their unique physicochemical properties, can not only induce apoptosis and damage DNA in cancer cells, but also significantly enhance the effects of anticancer drugs, which in turn may become a new approach to cancer treatment. It should be emphasized that the effectiveness of targeted drug delivery systems is also related to the structure, size, and shape of metal nanoparticles. However, despite the encouraging results obtained, further detailed studies in animal models are needed to better understand the molecular mechanisms associated with metal nanoparticles and their cytotoxic effects on various tumor cells.

## Footnotes

1
smart.servier.com
